# Morphology-Guided Surgical Management of Complex Traumatic Hip Fracture-Dislocations and Acetabular Fractures: A Six-Case Series Illustrating Morphology-Based Surgical Approaches

**DOI:** 10.7759/cureus.114457

**Published:** 2026-08-13

**Authors:** Noel S Singh, Manish Bisht, Raunaq Saxena

**Affiliations:** 1 Department of Orthopaedics, Graphic Era Institute of Medical Sciences, Dehradun, IND

**Keywords:** acetabular fracture, ganz safe surgical dislocation, kocher langenbeck approach, modified stoppa approach, traumatic hip dislocation

## Abstract

Traumatic hip fracture-dislocations and complex acetabular fractures are uncommon but severe injuries caused by high-energy trauma that require prompt diagnosis and individualized surgical management. Although emergency reduction is essential for hip dislocations, definitive treatment depends on fracture morphology. This case series illustrates how fracture morphology can guide individualized operative planning in complex traumatic hip and acetabular injuries.

We present six patients with distinct traumatic hip fracture-dislocations and acetabular fracture patterns, each managed using an individualized surgical approach according to the underlying fracture morphology. The first patient was a 26-year-old male with a central hip fracture-dislocation associated with fractures of the anterior column, quadrilateral surface, iliac blade, and an undisplaced posterior column, managed through the modified Stoppa approach following prophylactic ligation of the corona mortis. The second patient was a 19-year-old male who presented with a posterior hip dislocation associated with a Pipkin type II femoral head fracture and underwent fixation through the Ganz safe surgical dislocation approach with trochanteric flip osteotomy. The third patient was a 25-year-old male with a posterior hip dislocation associated with a posterior wall acetabular fracture, treated through the Kocher-Langenbeck approach using spring plates and posterior reconstruction plating. The fourth patient was a 38-year-old female with a complex both-column acetabular fracture (AO/OTA type 62-C3), managed with a combined anterior and posterior approach and sequential reconstruction of the anterior column, posterior column, and quadrilateral surface. The fifth patient was a 22-year-old male with a both-column acetabular fracture (AO/OTA type 62-C1), treated using the modified Stoppa approach with the first window of the ilioinguinal approach, allowing stable fixation of the pelvic brim, quadrilateral surface, and posterior column. The sixth patient was a 45-year-old male with an associated anterior and posterior column acetabular fracture managed through the Ganz safe surgical dislocation approach with trochanteric flip osteotomy, enabling direct visualization of the posterior acetabulum and anatomical reconstruction while preserving femoral head vascularity.

Computed tomography with three-dimensional reconstruction accurately defined fracture morphology and guided surgical planning. Surgical exposure was individualized according to fracture configuration, with anatomical reduction, stable fixation, satisfactory radiological alignment, and clinical recovery documented in all patients. This six-case series demonstrates that traumatic hip fracture-dislocations and complex acetabular fractures comprise a heterogeneous spectrum of injuries requiring individualized surgical management. Careful CT-based assessment and morphology-guided selection of the surgical approach facilitated appropriate exposure, anatomical reconstruction, and stable fixation in these cases. The modified Stoppa, Ganz safe surgical dislocation, Kocher-Langenbeck, combined anterior and posterior, and modified Stoppa with the first window of the ilioinguinal approaches each provided effective management for specific fracture patterns. These cases illustrate the feasibility of morphology-guided operative planning with satisfactory radiological and clinical outcomes.

## Introduction

Traumatic hip dislocations and complex acetabular fractures are uncommon but severe orthopedic injuries that usually result from high-energy trauma, most commonly road traffic accidents and falls from height. These injuries predominantly affect young adults and are considered orthopedic emergencies because delayed diagnosis or inappropriate management can lead to permanent disability, post-traumatic arthritis, avascular necrosis of the femoral head, and chronic functional impairment [1-5]. Hip dislocations are classified as anterior, posterior, or central according to the position of the femoral head relative to the acetabulum, with posterior dislocations being the most common type [5,6]. Acetabular fractures, particularly those involving the anterior and posterior columns or the quadrilateral surface, present additional challenges owing to disruption of pelvic stability and the complexity of achieving anatomical reconstruction [2,3].

Early reduction of a dislocated hip remains one of the most important determinants of prognosis. Reduction performed within six hours of injury has been shown to reduce the risk of avascular necrosis of the femoral head, whereas delayed reduction increases the likelihood of complications such as avascular necrosis, post-traumatic arthritis, hip stiffness, and chronic pain [4,5,7]. Although isolated hip dislocations may be managed successfully with closed reduction, associated acetabular or femoral head fractures frequently necessitate open reduction and internal fixation to restore joint congruity, stability, and function [2,4,8]. Similarly, complex acetabular fractures without hip dislocation often require meticulous surgical reconstruction because anatomical reduction is one of the strongest predictors of long-term functional outcome.

Fracture morphology is the principal determinant of the surgical approach. The Judet-Letournel classification remains the standard system for classifying acetabular fractures into elementary and associated patterns, while the AO/OTA classification provides a comprehensive description of fracture complexity and guides operative planning [9]. Modern multidetector computed tomography with three-dimensional reconstruction has significantly improved the evaluation of these injuries by accurately defining fracture morphology, identifying intra-articular fragments, assessing column involvement, and facilitating selection of the most appropriate surgical exposure and fixation strategy [9]. Consequently, successful management depends not only on timely reduction of hip dislocations but also on choosing the surgical approach that best addresses the underlying fracture configuration.

In this case series, we present six patients with distinct traumatic hip fracture-dislocations and complex acetabular fractures to illustrate how fracture morphology and CT findings guided the selection of individualized surgical approaches. We also describe the radiological and clinical outcomes following morphology-guided surgical management.

## Materials and methods

This retrospective case series included six patients with complex traumatic hip fracture-dislocations and acetabular fractures who underwent surgical management at the Department of Orthopedics, Graphic Era Institute of Medical Sciences, Dehradun, India, between January 2025 and December 2025. Patients were selected to represent distinct fracture patterns in which preoperative CT assessment influenced the choice of surgical approach. Clinical records, radiographs, CT scans with three-dimensional reconstructions, operative notes, and postoperative records were retrospectively reviewed.

Patients were included if they had a complex traumatic hip fracture-dislocation or acetabular fracture requiring operative management, adequate preoperative CT imaging for assessment of fracture morphology, and sufficient clinical and radiological records for postoperative evaluation. Patients with incomplete clinical or imaging records were excluded.

All patients underwent preoperative evaluation with anteroposterior pelvic radiographs and CT imaging with three-dimensional reconstruction to define fracture morphology and guide surgical planning. The surgical approach was individualized according to the fracture configuration. The modified Stoppa approach was used for the central acetabular fracture-dislocation with anterior column and quadrilateral surface involvement. The Ganz safe surgical dislocation approach was used for the Pipkin type II femoral head fracture and for a selected associated anterior and posterior column acetabular fracture requiring direct visualization of the posterior acetabulum. The Kocher-Langenbeck approach was used for the posterior wall acetabular fracture associated with hip dislocation. Complex both-column acetabular fractures were managed using either a combined anterior and posterior approach or the modified Stoppa approach with the first window of the ilioinguinal approach. Appropriate internal fixation techniques were used to achieve anatomical reduction and stable fixation.

Postoperative follow-up included clinical and radiological assessment at routine postoperative visits based on the available clinical records. Outcomes were assessed based on fracture reduction, hip congruity, implant stability, fracture union, range of motion, pain, ambulation, and overall functional recovery. No standardized functional outcome score, such as the Harris Hip Score or Western Ontario and McMaster Universities Osteoarthritis Index (WOMAC), was used.

According to the institutional policy of Graphic Era Institute of Medical Sciences, Dehradun, India, this case series was exempt from formal Institutional Ethics Committee review. Written informed consent for publication of the clinical details and accompanying images was obtained from all patients. All identifying information was removed to maintain patient confidentiality.

## Results

Case 1: Central hip fracture-dislocation with complex acetabular fracture managed through the modified Stoppa approach

**Figure 1:**
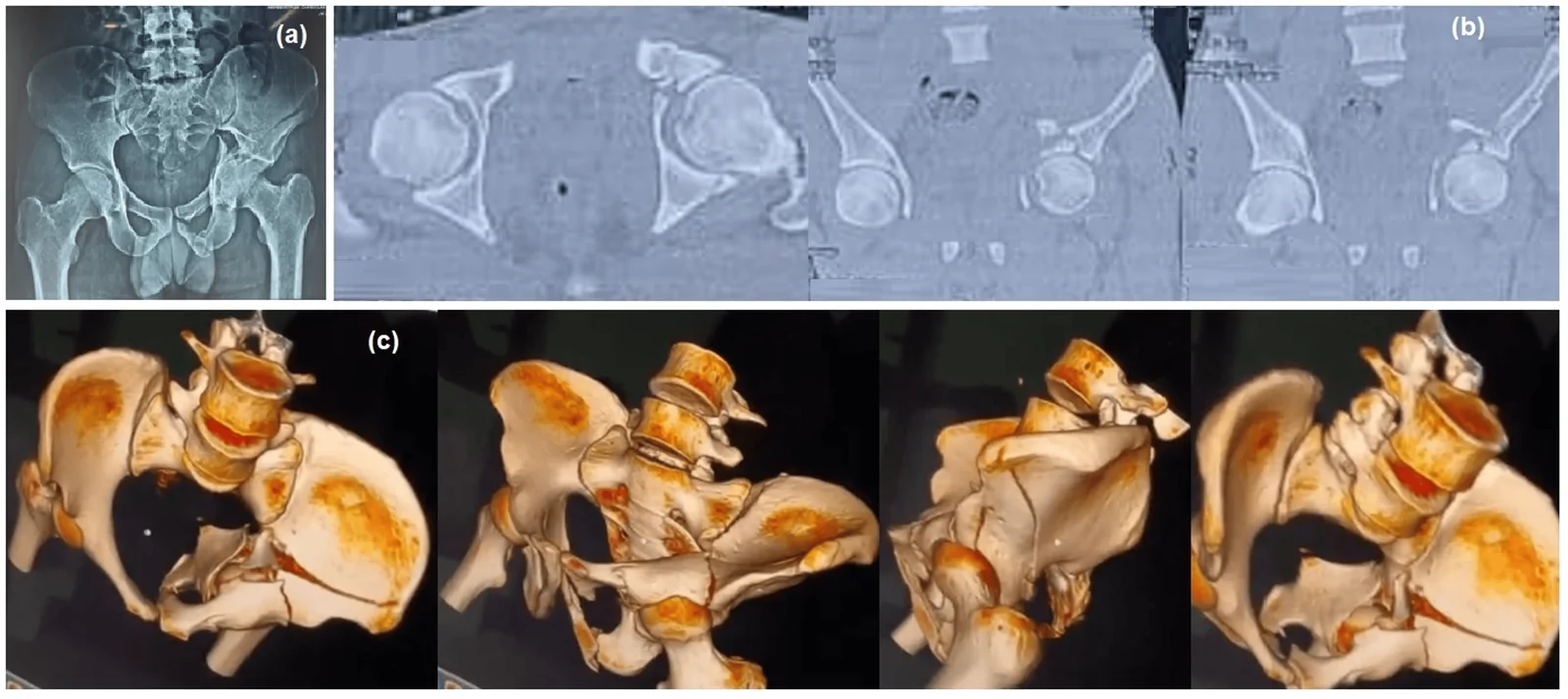
Case 1: Preoperative evaluation. (a) Anteroposterior pelvic radiograph demonstrating a central hip fracture-dislocation with a complex acetabular fracture. (b) Axial CT image showing medial displacement of the femoral head with involvement of the quadrilateral surface and anterior column. (c) Three-dimensional CT reconstruction demonstrating the complete fracture morphology, including fractures of the anterior column, quadrilateral surface, posterior column, and iliac blade.

A 26-year-old male patient presented following a high-energy injury by fall from height, with severe pain around the left hip and inability to bear weight. Clinical examination revealed a painful, restricted left hip without any distal neurovascular deficit. Initial anteroposterior pelvic radiographs demonstrated a central fracture-dislocation of the left hip. CT with three-dimensional reconstruction confirmed a displaced anterior column acetabular fracture associated with a quadrilateral surface fracture and an iliac blade fracture, while the posterior column fracture remained undisplaced (Figure 1).

**Figure 2:**
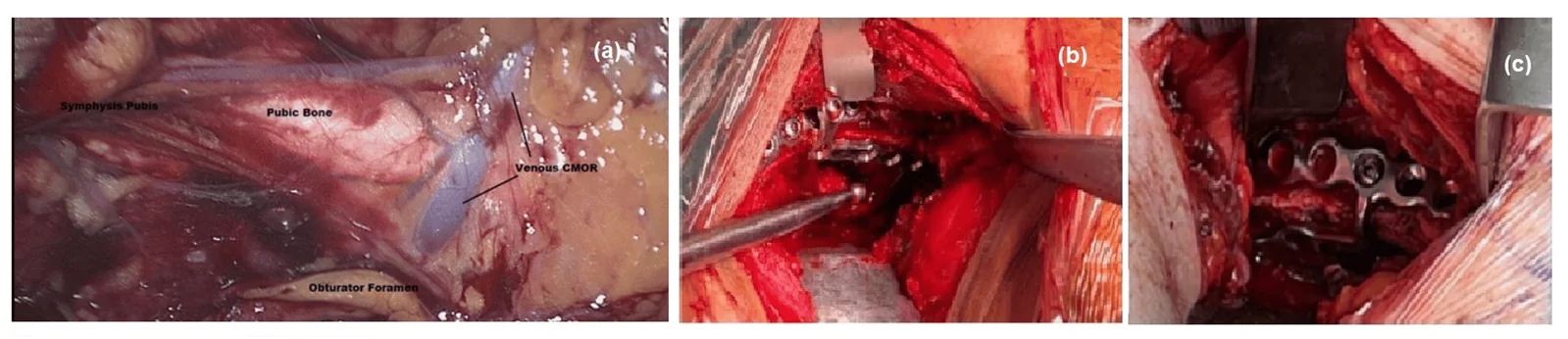
Case 1: Intraoperative management. (a) Identification of the corona mortis before prophylactic ligation. (b) Modified Stoppa approach demonstrating reduction of the anterior column and quadrilateral surface. (c) Definitive fixation using anterior column plating and quadrilateral buttress fixation.

Considering the fracture morphology, definitive fixation through the modified Stoppa approach was planned. As the fracture extended close to the superior pubic ramus, prophylactic identification and ligation of the corona mortis was performed before orthopedic exposure to minimize the risk of intrapelvic vascular injury during dissection (Figure 2).

Following vascular control, the patient was positioned supine, and the acetabulum was exposed through the modified Stoppa approach. The displaced anterior column fracture was anatomically reduced and stabilized using reconstruction plate fixation. The quadrilateral surface was buttressed to restore the medial acetabular wall, and the associated iliac blade fracture was fixed with cortical screws. As the posterior column fracture was minimally displaced and remained stable after fixation of the anterior column construct, no additional posterior approach or fixation was required.

**Figure 3:**
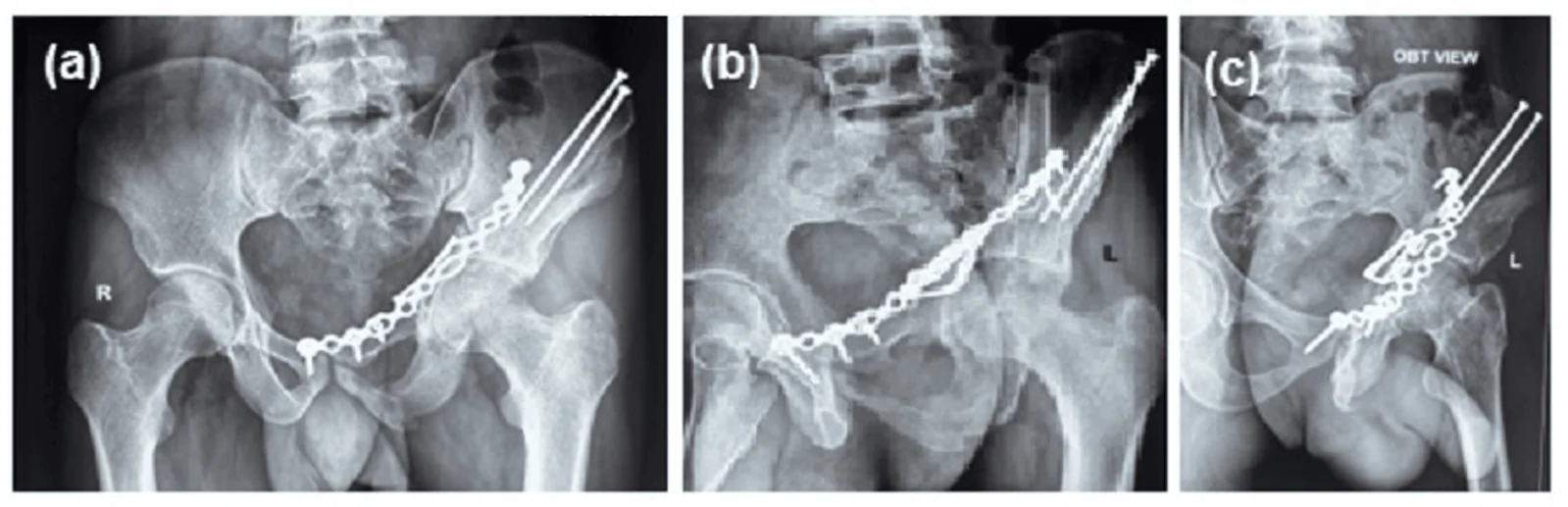
Case 1: Immediate postoperative assessment. (a) Immediate postoperative anteroposterior pelvic radiograph. (b) Immediate postoperative iliac oblique (Judet) radiograph. (c) Immediate postoperative obturator oblique (Judet) radiograph.

**Figure 4:**
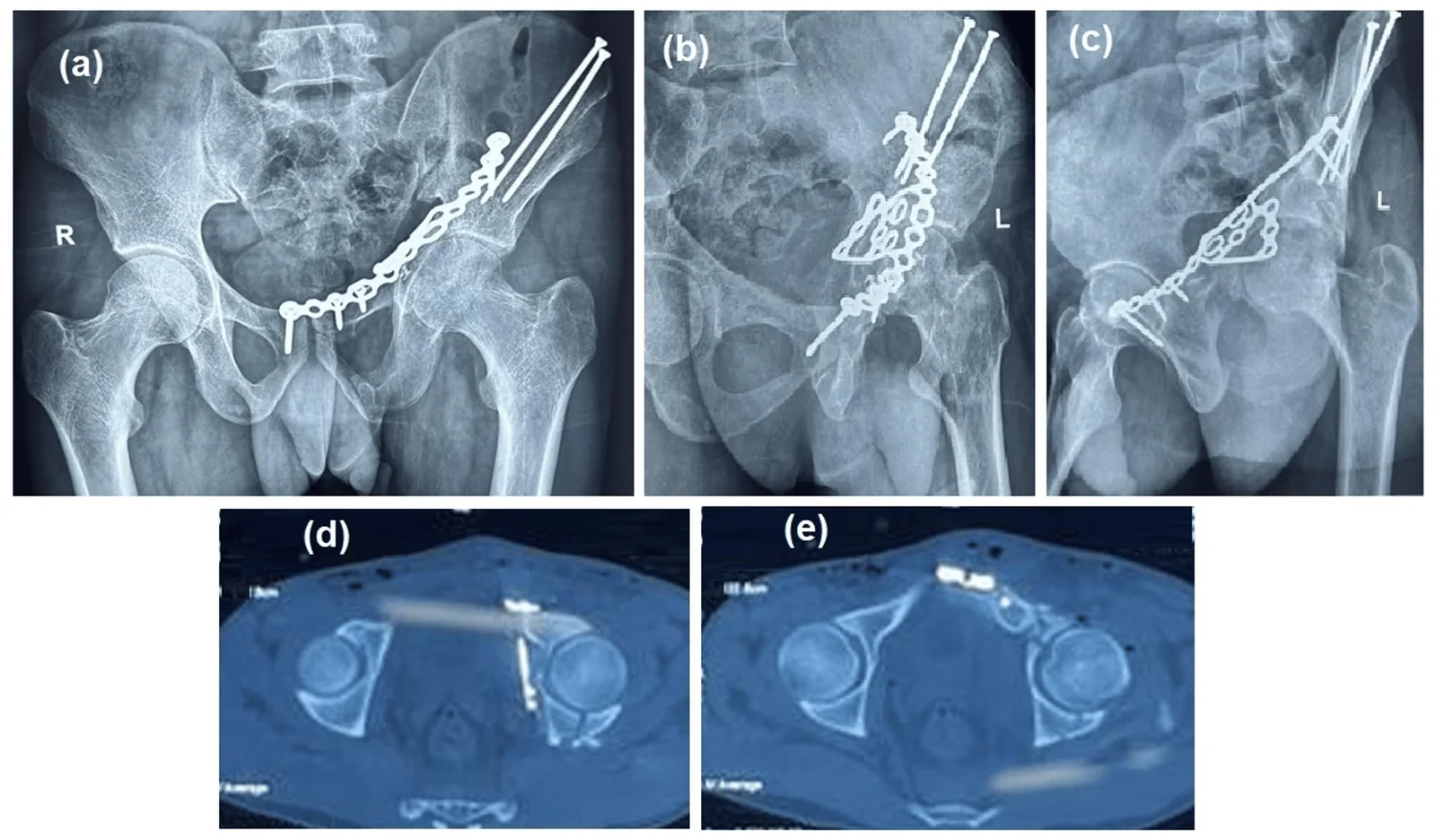
Case 1: Radiological follow-up. (a) Follow-up anteroposterior pelvic radiograph. (b) Follow-up iliac oblique radiograph. (c) Follow-up obturator oblique radiograph. (d) Follow-up axial CT image. (e) Follow-up axial CT image demonstrating maintained reduction and stable implant position.

Immediate postoperative radiographs demonstrated satisfactory anatomical reduction of the acetabulum with stable fixation of the anterior column, quadrilateral surface, and iliac blade fractures (Figure 3). Follow-up radiographs and CT scans confirmed maintained reduction, progressive fracture union, stable implant position, and healing of the untreated posterior column fracture without secondary displacement (Figure 4).

**Figure 5:**
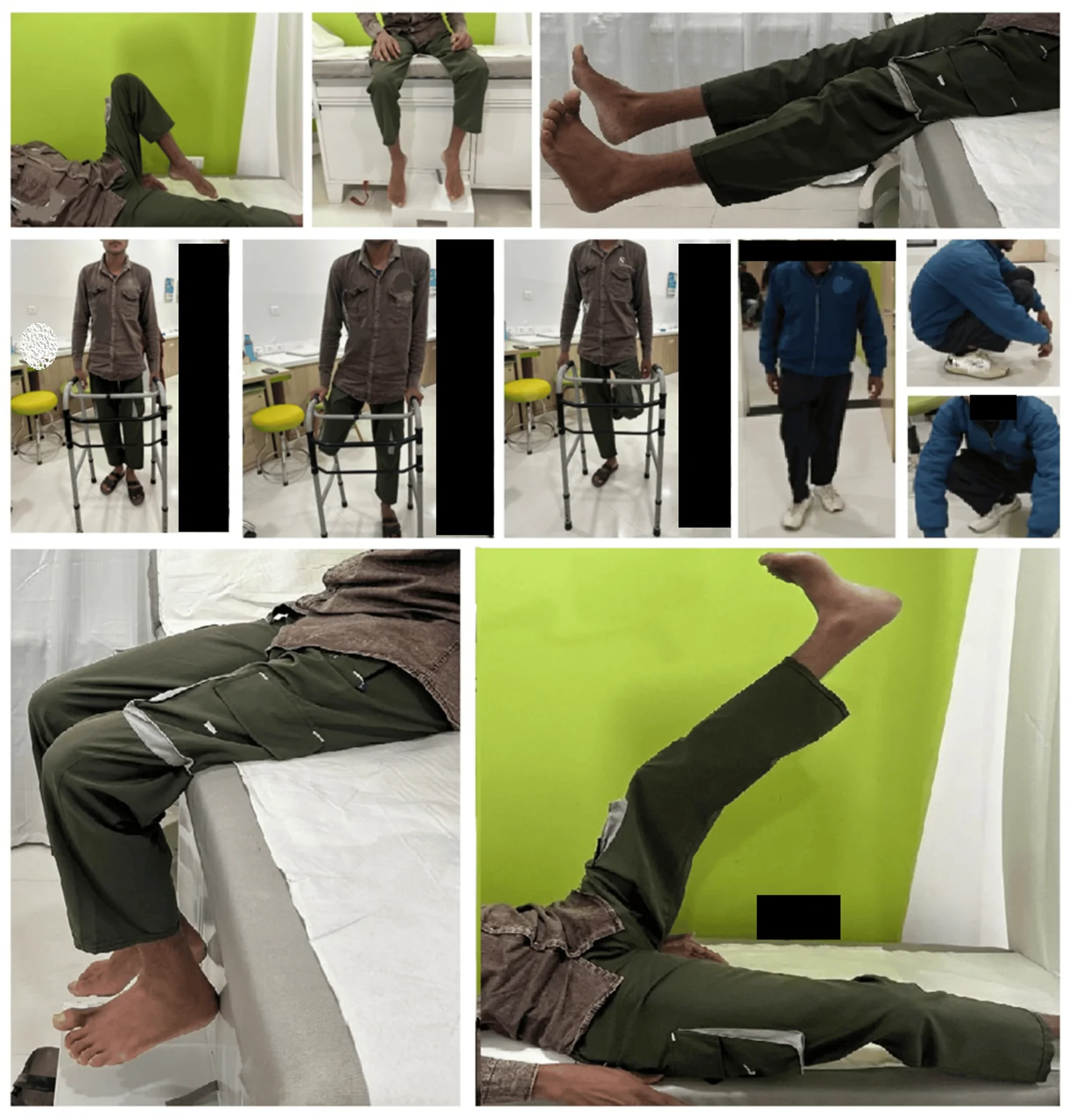
Case 1: Clinical photographs demonstrating satisfactory hip range of motion, independent ambulation, and good functional recovery at the latest follow-up.

At the most recent follow-up, the patient was ambulatory with satisfactory hip range of motion and good functional recovery. Clinical photographs demonstrated maintained hip congruity without implant failure, loss of reduction, or secondary displacement (Figure 5).

Learning Point

Central hip fracture-dislocations with predominant anterior column and quadrilateral surface involvement can be effectively managed through the modified Stoppa approach. In selected cases, prophylactic ligation of the corona mortis may facilitate safer intrapelvic dissection and allow stable anatomical reconstruction with satisfactory functional recovery.

Case 2: Posterior hip dislocation with Pipkin type II femoral head fracture managed through the Ganz safe surgical dislocation approach

**Figure 6:**
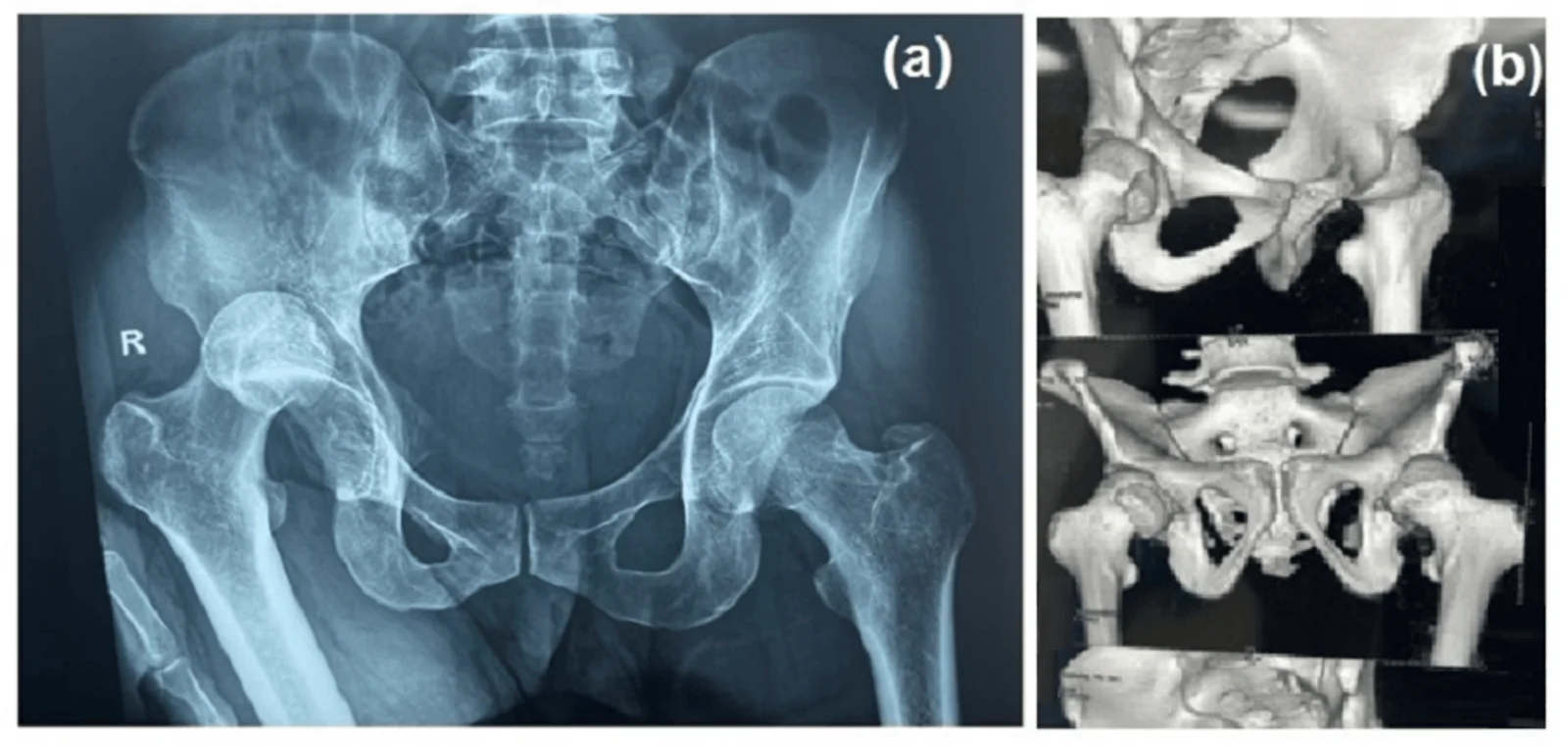
Case 2: Preoperative evaluation. (a) Anteroposterior pelvic radiograph at presentation demonstrating posterior dislocation of the right hip. (b) Three-dimensional CT reconstruction obtained after reduction demonstrating a Pipkin type II femoral head fracture involving the weight-bearing surface.

A 19-year male patient presented following a high-energy traumatic injury from road accident with severe pain in the affected hip and inability to bear weight. Clinical examination demonstrated a painful, deformed hip with marked restriction of movement, while distal neurovascular examination was normal. Emergency radiographs revealed a posterior dislocation of the hip. Following urgent closed reduction, CT demonstrated a displaced Pipkin type II femoral head fracture involving the weight-bearing surface without associated femoral neck fracture (Figure 6).

Considering the displaced intra-articular fracture involving the weight-bearing surface of the femoral head, open reduction and internal fixation through the Ganz safe surgical dislocation approach was planned. This approach was selected to provide complete visualization of the femoral head while preserving its vascular supply.

**Figure 7:**
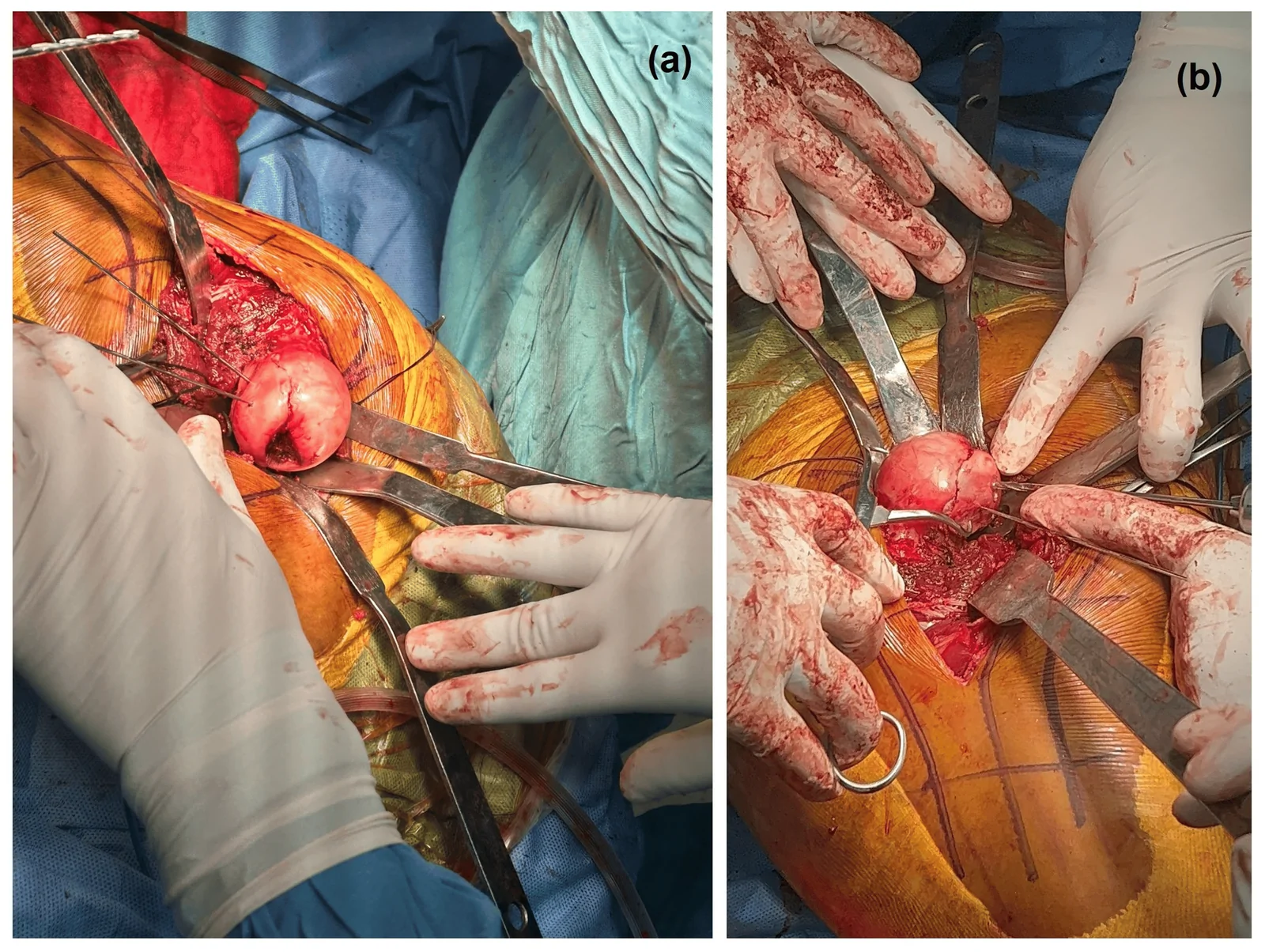
Case 2: Intraoperative management. (a) Surgical dislocation of the hip demonstrating the displaced femoral head fracture. (b) Anatomical reduction and fixation of the femoral head fracture using Herbert headless compression screws.

The patient was positioned in the lateral decubitus position, and a trochanteric flip osteotomy was performed to safely dislocate the hip. Following surgical dislocation, the femoral head fracture was anatomically reduced and stabilized using Herbert headless compression screws (Figure 7). The greater trochanteric osteotomy was subsequently reduced and fixed using cancellous screws.

**Figure 8:**
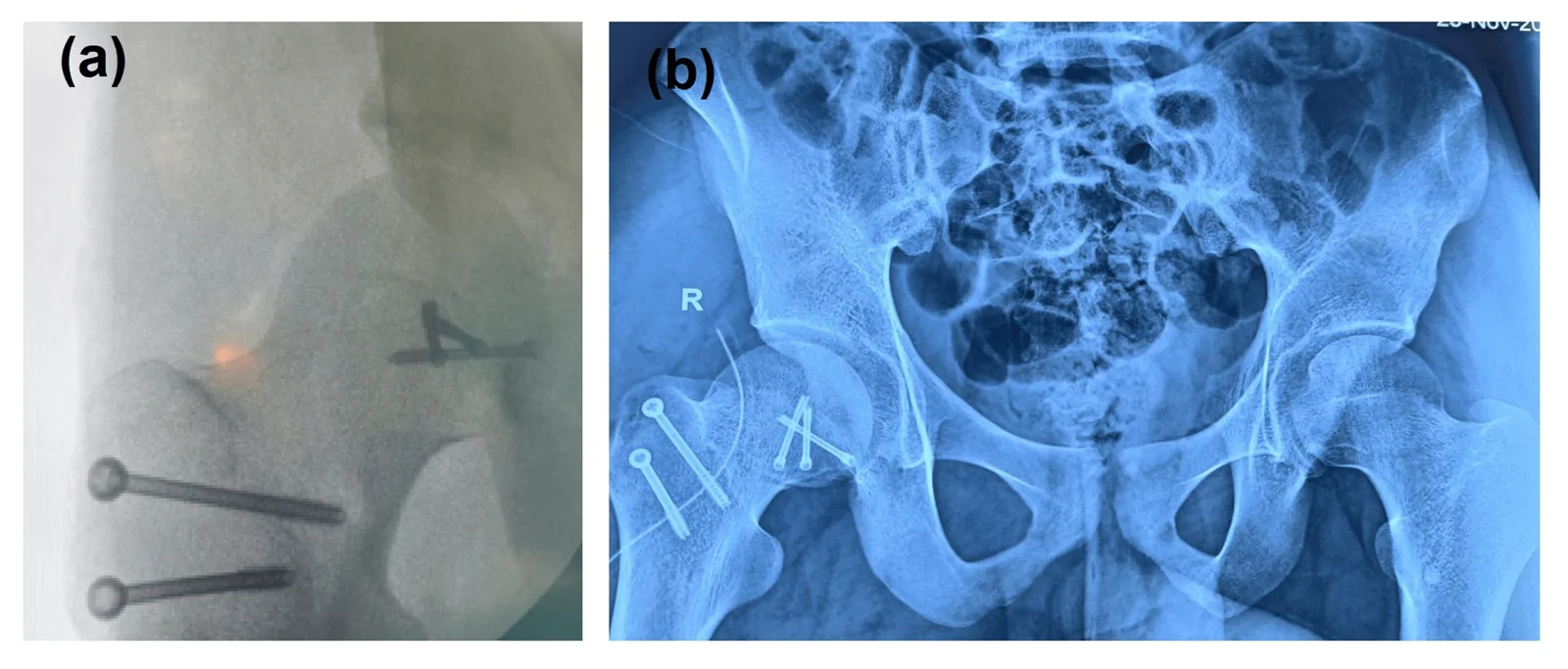
Case 2: Intraoperative fluoroscopy and immediate postoperative assessment. (a) Intraoperative fluoroscopic image demonstrating anatomical reduction of the femoral head fracture and fixation of the greater trochanteric osteotomy. (b) Immediate postoperative anteroposterior radiograph confirming concentric reduction of the hip and satisfactory implant position.

Intraoperative fluoroscopy confirmed satisfactory reduction of the femoral head and appropriate implant placement. The greater trochanteric osteotomy was securely fixed with cancellous screws, restoring hip stability (Figure 8).

At the latest follow-up, radiographs demonstrated maintained hip congruity with union of the greater trochanteric osteotomy and stable fixation of the femoral head fracture. The patient had a painless hip with satisfactory range of motion, independent ambulation, and no radiographic evidence of fixation failure or secondary displacement.

Learning Point

The Ganz safe surgical dislocation approach provides circumferential visualization of the femoral head while preserving its vascular supply, facilitating anatomical reduction and stable fixation of Pipkin type II femoral head fractures.

Case 3: Posterior hip dislocation with posterior wall acetabular fracture managed through the Kocher-Langenbeck approach

**Figure 9:**
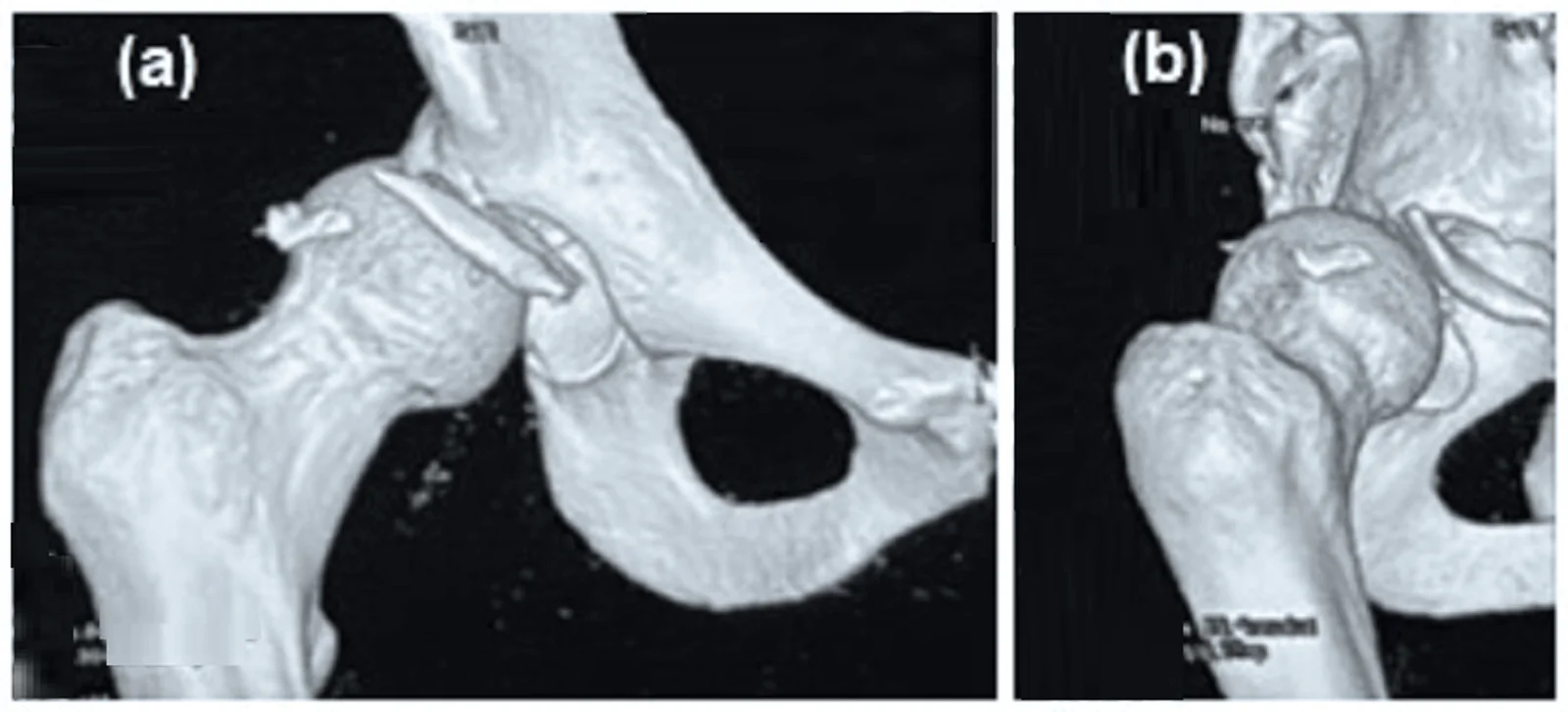
Case 3: Preoperative evaluation. (a) Three-dimensional CT reconstruction demonstrating posterior hip dislocation associated with a posterior wall acetabular fracture. (b) Three-dimensional CT reconstruction showing the incarcerated posterior wall fragment within the hip joint, preventing concentric reduction.

A 25-year-old male patient presented following a high-energy traumatic injury by road accident, with severe pain in the right hip and an inability to bear weight. Clinical examination revealed a painful, deformed hip with marked restriction of movement, while distal neurovascular examination was unremarkable. CT with three-dimensional reconstruction demonstrated a posterior hip dislocation associated with a posterior wall acetabular fracture. The displaced posterior wall fragment was incarcerated within the hip joint, preventing concentric reduction and resulting in persistent hip instability (Figure 9).

**Figure 10:**
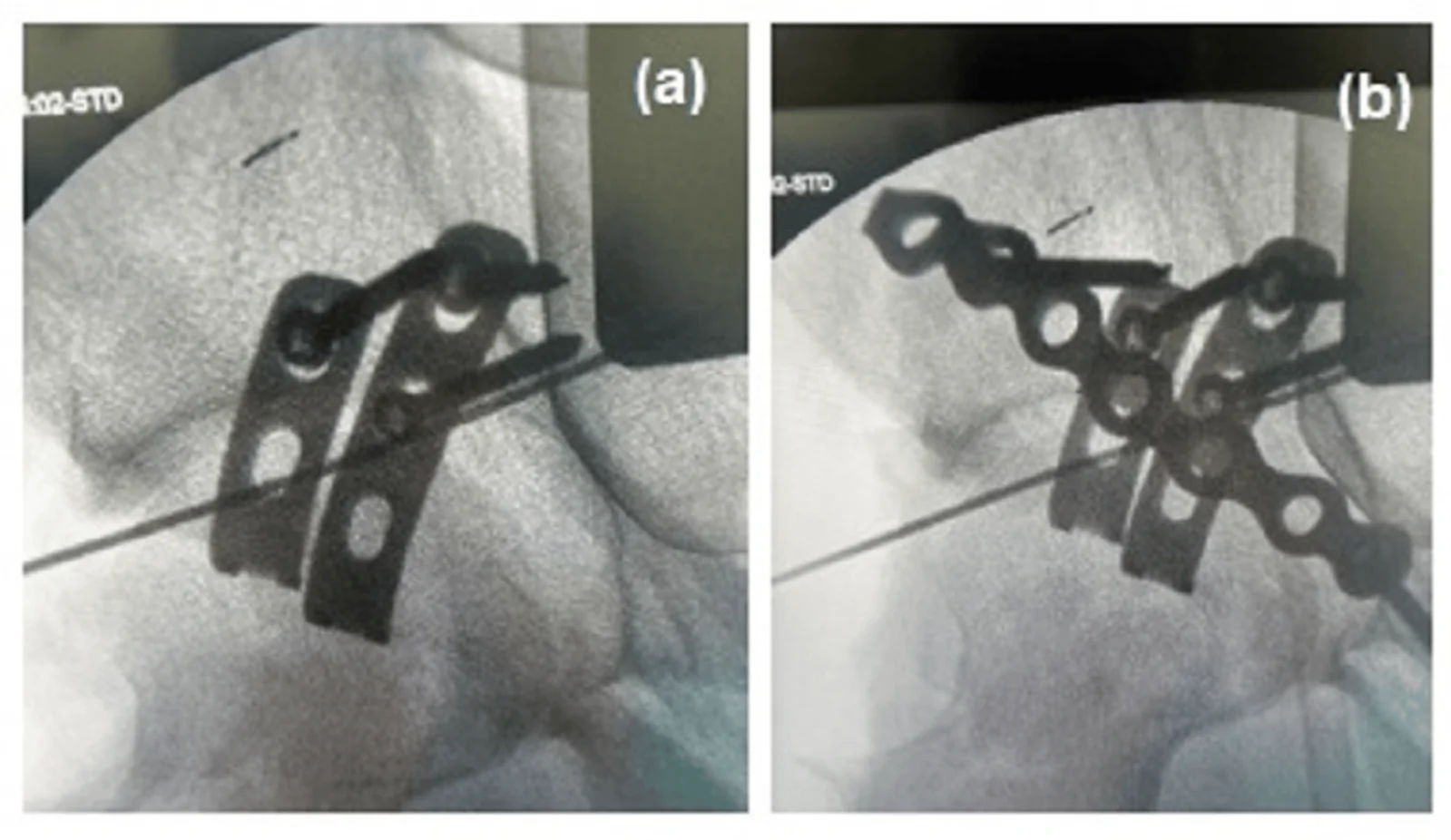
Case 3: Intraoperative management. (a) Intraoperative fluoroscopic image demonstrating anatomical reduction and spring plate fixation of the posterior wall fragment. (b) Intraoperative fluoroscopic image demonstrating definitive posterior reconstruction plate fixation providing stable buttress support.

Considering the irreducible posterior wall fragment and unstable hip joint, open reduction and internal fixation through the Kocher-Langenbeck approach was performed. The posterior wall fragment was identified, anatomically reduced, and provisionally stabilized. Spring plates were applied to secure the osteochondral fragments, followed by placement of a posterior reconstruction plate to buttress the posterior wall and restore acetabular stability (Figure 10).

**Figure 11:**
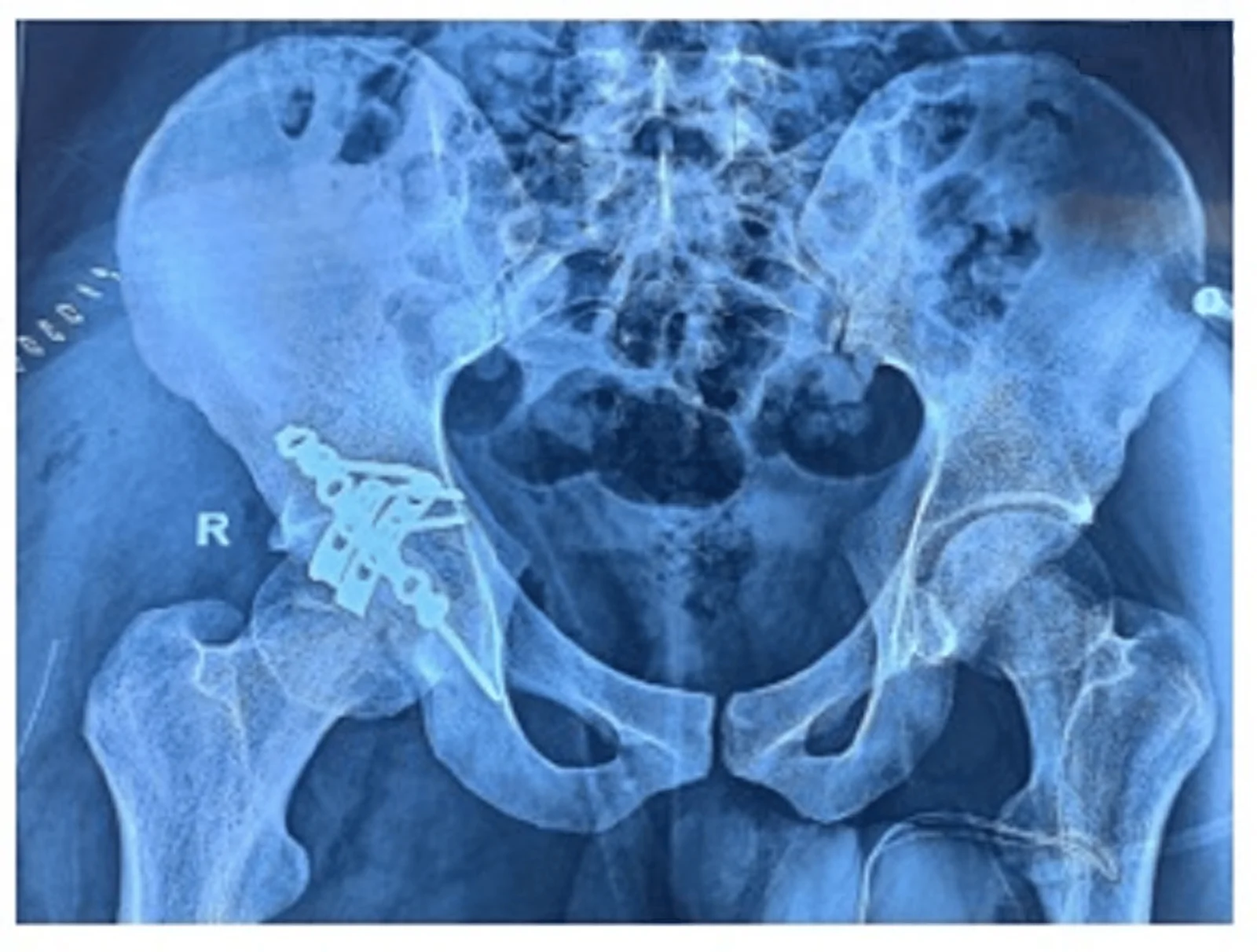
Case 3: Immediate postoperative anteroposterior pelvic radiograph demonstrating concentric reduction of the hip joint with stable spring plate and posterior reconstruction plate fixation.

Immediate postoperative radiographs demonstrated concentric reduction of the hip joint with satisfactory restoration of the posterior acetabular wall and stable implant position. At the latest follow-up, the patient had a stable, painless hip with satisfactory range of motion and independent ambulation. No radiographic evidence of implant failure or loss of reduction was observed (Figure 11).

Learning Point

Posterior wall acetabular fractures associated with hip dislocation may become irreducible when the fracture fragment is incarcerated within the joint. The Kocher-Langenbeck approach provides direct visualization of the posterior acetabulum, allowing anatomical reduction and stable fixation with spring plates and buttress plating to restore hip stability.

Case 4: Both column acetabular fracture managed through combined anterior and posterior approaches

**Figure 12:**
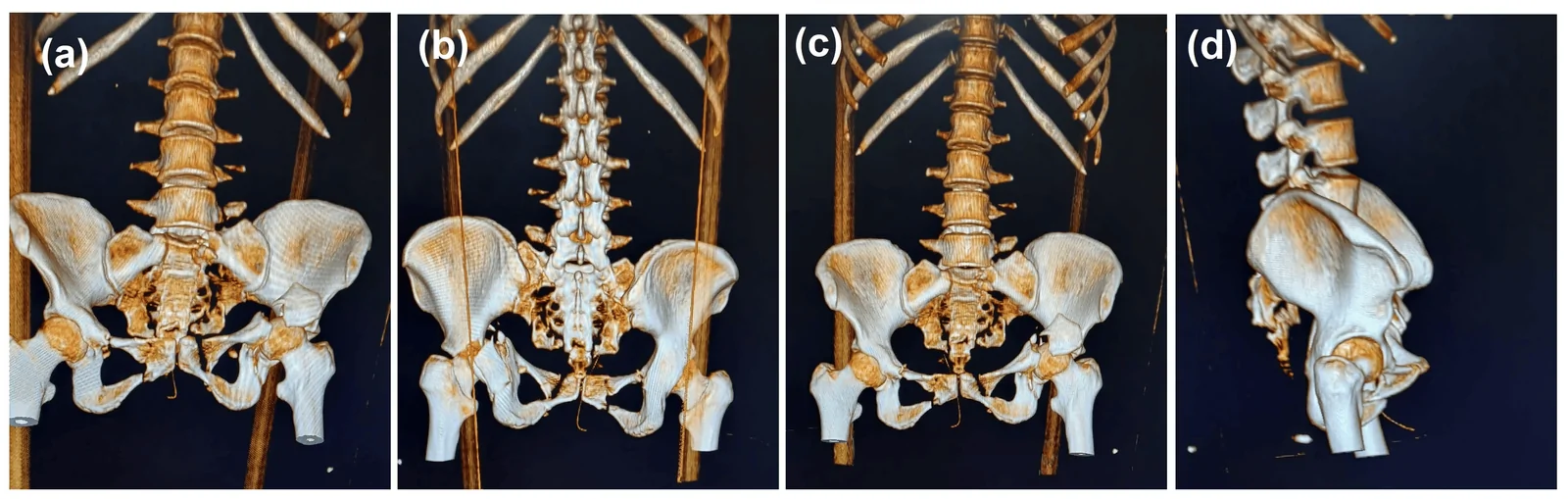
Case 4: Preoperative evaluation. (a) Left anterior oblique three-dimensional CT reconstruction demonstrating the displaced both-column acetabular fracture involving the anterior and posterior columns. (b) Anterior three-dimensional CT reconstruction showing disruption of the pelvic brim and acetabular architecture. (c) Right anterior oblique three-dimensional CT reconstruction illustrating displacement of the anterior and posterior columns and involvement of the quadrilateral surface. (d) Lateral three-dimensional CT reconstruction of the affected hemipelvis demonstrating the complex fracture morphology and extent of displacement.

A 38-year-old female presented following a fall from a height of approximately 40 feet, with severe pain around the left hip and an inability to bear weight. Clinical examination revealed painful restriction of hip movements without a distal neurovascular deficit. CT with three-dimensional reconstruction demonstrated a displaced both-column acetabular fracture (AO/OTA type 62-C3) involving the anterior column, posterior column, quadrilateral surface, and superior pubic ramus (Figure 12).

**Figure 13:**
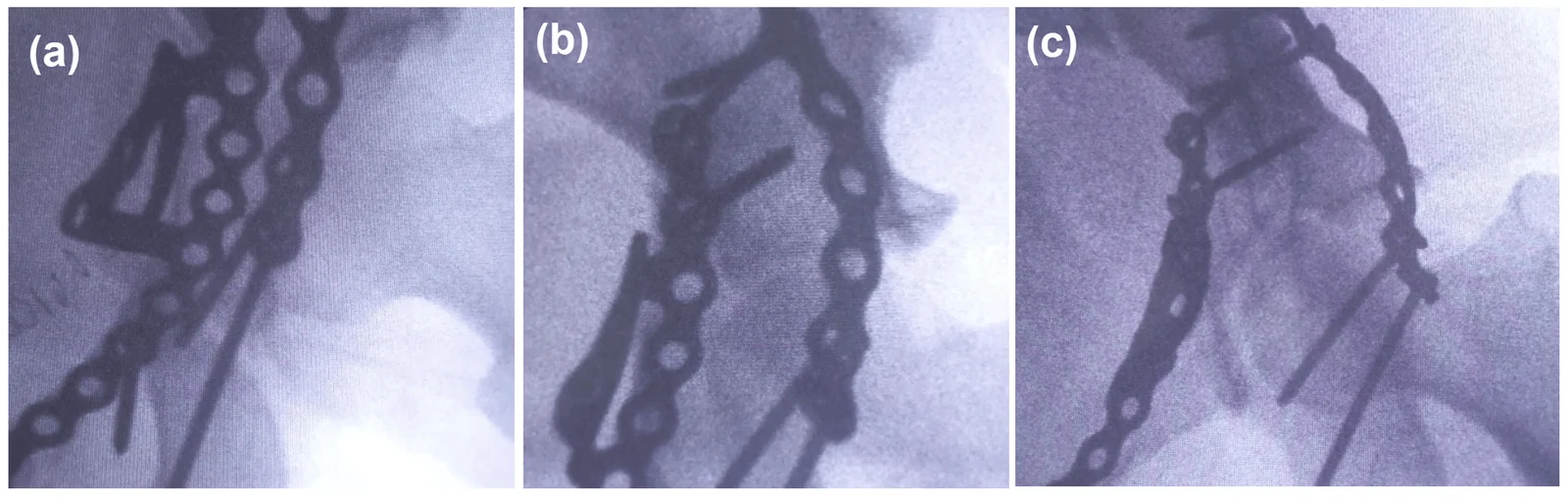
Case 4: Intraoperative management. (a) Intraoperative fluoroscopic iliac oblique view demonstrating reduction of the anterior column and provisional fixation with reconstruction plates and screws. (b) Intraoperative fluoroscopic obturator oblique view confirming anatomical reduction of the acetabular columns and restoration of the articular surface. (c) Intraoperative fluoroscopic anteroposterior pelvic view demonstrating final reconstruction plate and screw fixation with satisfactory alignment of the both-column acetabular fracture following the combined anterior and posterior approach.

Considering the complex fracture morphology, definitive fixation was performed using a combined anterior and posterior approach. Through the anterior intrapelvic approach, the bladder was protected, the corona mortis was identified and ligated, and the iliacus was elevated to expose the pelvic brim, quadrilateral surface, superior pubic ramus, and internal iliac fossa. Reduction was performed sequentially by first restoring the anterior column using reduction clamps and temporary Kirschner wire fixation, followed by reduction of the posterior column using pelvic reduction clamps and Schanz pin joystick manipulation. The medially displaced quadrilateral surface was reduced and buttressed, and definitive fixation was achieved with a 3.5-mm reconstruction plate along the pelvic brim, followed by posterior column fixation through the posterior approach (Figure 13).

Intraoperative fluoroscopy confirmed satisfactory anatomical reduction of both acetabular columns with stable implant placement. The combined anterior and posterior approach provided adequate visualization for sequential reduction and stable fixation of the complex fracture pattern.

Learning Point

Both-column acetabular fractures require meticulous CT-based preoperative assessment and morphology-guided operative planning. Sequential reduction of the anterior column, posterior column, and quadrilateral surface through combined anterior and posterior approaches facilitates anatomical reconstruction and stable fixation in AO/OTA type 62-C3 fractures.

Case 5: Both column acetabular fracture managed through the modified Stoppa approach with first window of the ilioinguinal approach

**Figure 14:**
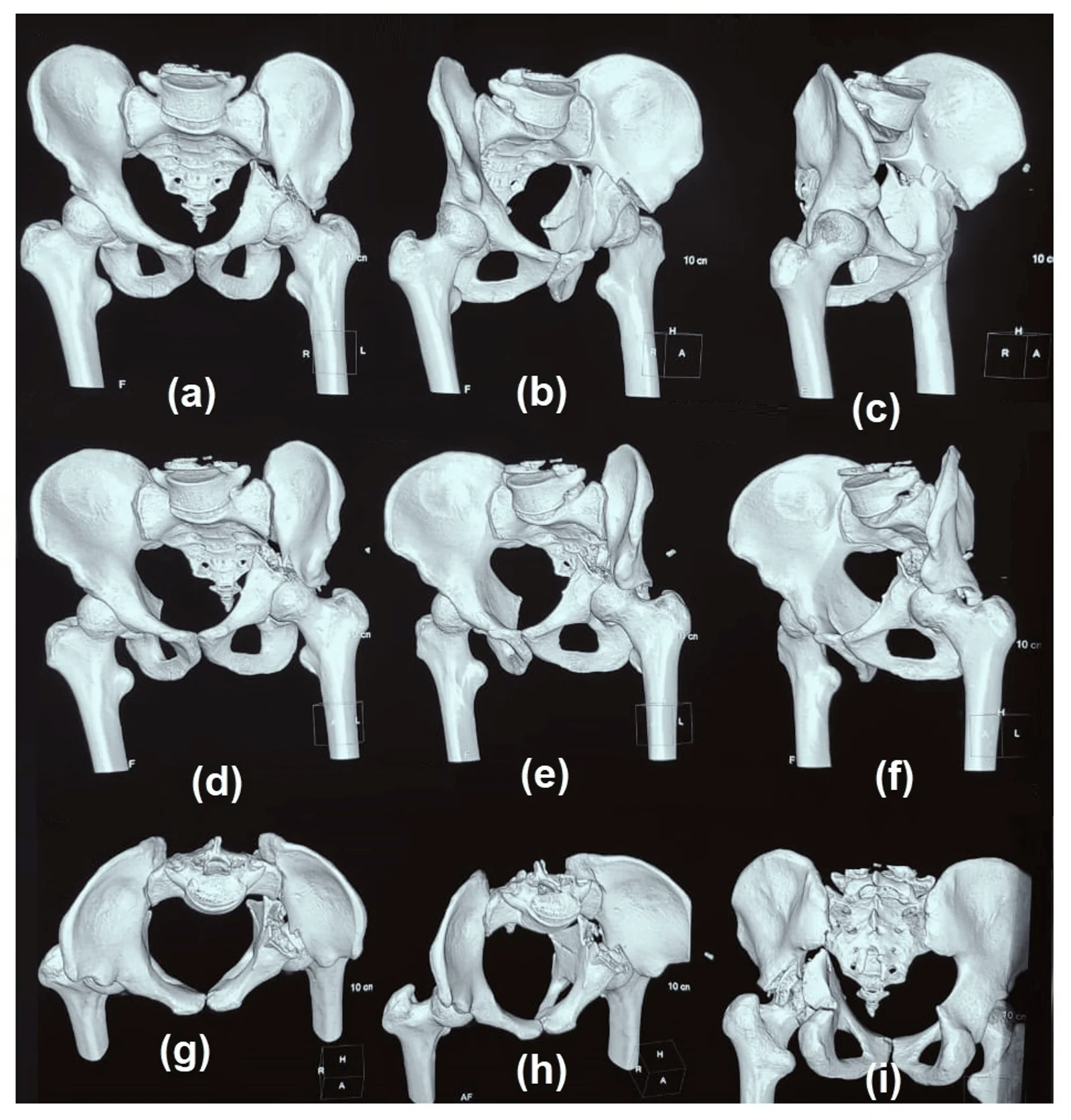
Case 5: Preoperative evaluation. (a) Anterior three-dimensional CT reconstruction demonstrating the displaced both-column acetabular fracture. (b) Left anterior oblique view illustrating disruption of the anterior and posterior columns with displacement of the acetabular articular segment. (c) Left lateral oblique view demonstrating the relationship between the anterior and posterior column fractures. (d) Anteroposterior view highlighting displacement of the acetabular fragment and disruption of the pelvic brim. (e) Left oblique view demonstrating quadrilateral surface involvement and medial displacement of the acetabular fragment. (f) Lateral view illustrating the posterior extent of the fracture and acetabular displacement. (g) Inferior pelvic view demonstrating complete separation of the acetabular fragment from the axial skeleton. (h) Inferolateral oblique view further illustrating the floating acetabular fragment and complex fracture configuration. (i) Posterior three-dimensional CT reconstruction demonstrating disruption of the posterior column and posterior pelvic anatomy.

A 22-year-old male presented following a fall from height with severe left hip pain and inability to bear weight. Clinical examination revealed painful restriction of hip movements without distal neurovascular deficit. Initial anteroposterior pelvic radiographs demonstrated a displaced acetabular fracture. CT with three-dimensional reconstruction confirmed a both-column acetabular fracture (AO/OTA type 62-C1; Letournel both-column fracture) with complete separation of the anterior and posterior columns from the axial skeleton, displacement of the quadrilateral surface, and involvement of the iliopubic and ilioischial columns (Figure 14).

**Figure 15:**
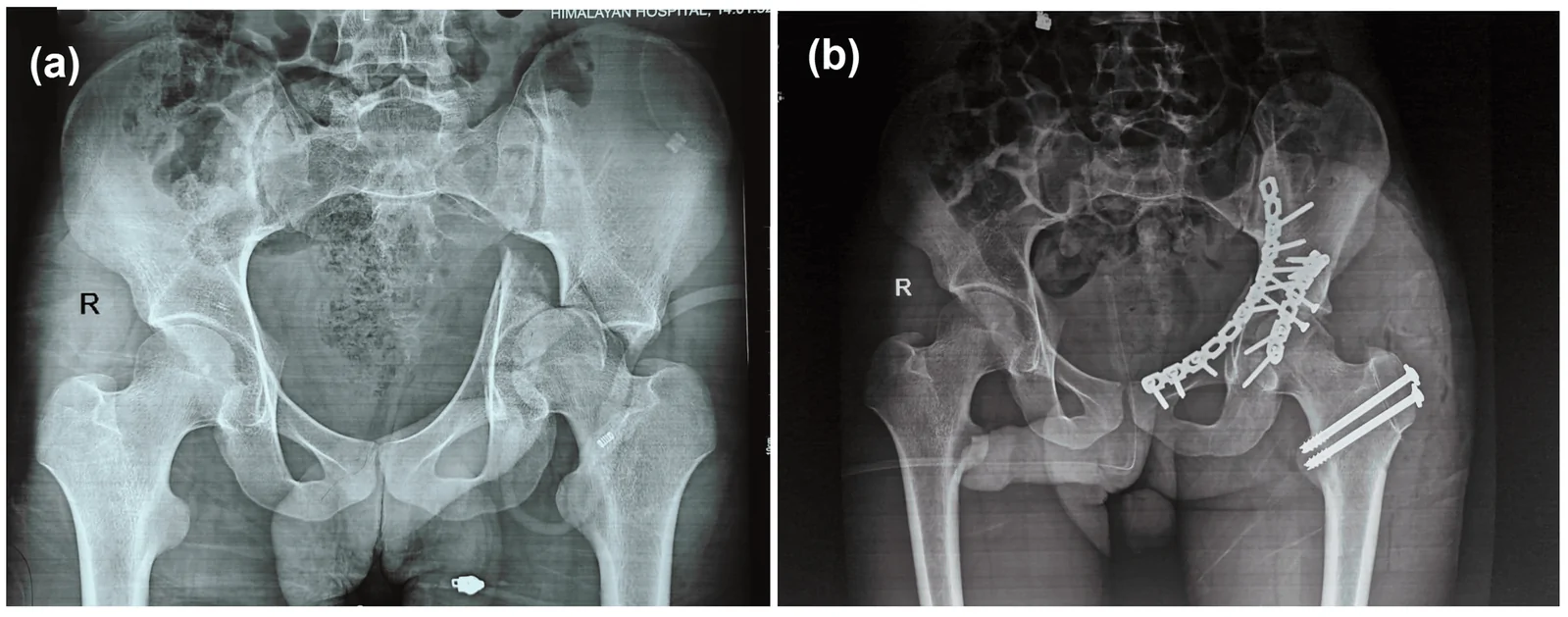
Case 5: Immediate postoperative assessment. (a) Preoperative anteroposterior pelvic radiograph demonstrating a displaced left both-column acetabular fracture. (b) Immediate postoperative anteroposterior pelvic radiograph demonstrating anatomical reduction and stable fixation using pelvic brim plating, iliac wing fixation, and posterior column screws.

Considering the fracture morphology, definitive fixation was performed through the modified Stoppa approach with an additional first window of the ilioinguinal approach. The anterior intrapelvic approach provided direct visualization of the pelvic brim and quadrilateral surface, while the lateral iliac window facilitated reduction and fixation of the iliac wing. Anatomical reduction was achieved through sequential reduction of the anterior and posterior columns, followed by buttress fixation of the quadrilateral surface. Definitive fixation was performed using a precontoured reconstruction plate along the pelvic brim with supplementary iliac fixation and posterior column screws inserted through the anterior approach (Figure 15).

Postoperative radiographs demonstrated satisfactory restoration of acetabular congruity with stable implant position. The patient had an uneventful postoperative recovery and was discharged on a structured rehabilitation protocol.

Learning Point

Both-column acetabular fractures require detailed CT-based assessment to define fracture morphology and guide surgical planning. The modified Stoppa approach combined with the first window of the ilioinguinal approach provides excellent exposure for anatomical reduction and stable fixation of AO/OTA type 62-C1 fractures while facilitating quadrilateral surface buttressing and posterior column fixation.

Case 6: Associated anterior and posterior column acetabular fracture managed through Ganz safe surgical dislocation with trochanteric flip osteotomy

A 45-year-old male presented seven days after sustaining a high-energy road traffic accident following a skid and fall from a two-wheeler. He complained of severe pain around the right hip and inability to bear weight. Clinical examination revealed tenderness over the right hip with painful restriction of hip movements. Pelvic compression and distraction tests were positive. Mild swelling was present around the hip without any open wound. Distal motor, sensory, and vascular examination was normal.

**Figure 16:**
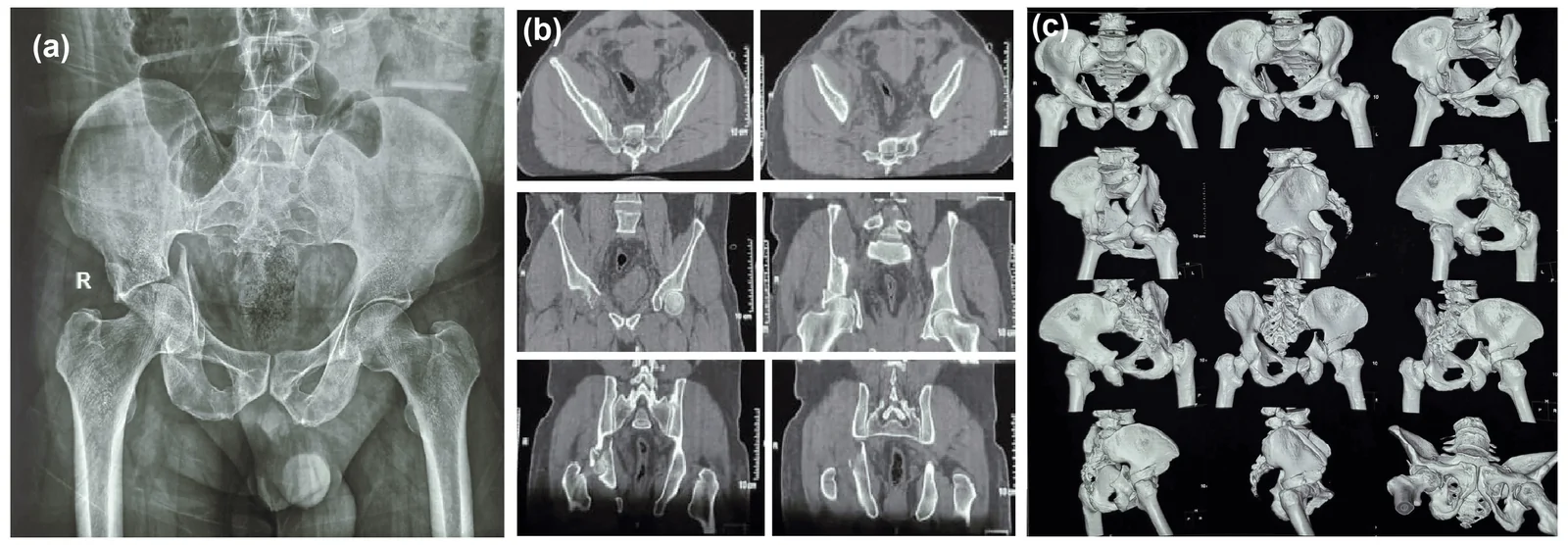
Case 6: Preoperative evaluation. (a) Anteroposterior pelvic radiograph demonstrating a displaced right acetabular fracture. (b) Representative axial and coronal computed tomography images demonstrating fractures involving the anterior and posterior acetabular columns with posterior column displacement. (c) Three-dimensional computed tomography reconstructions illustrating the overall fracture configuration from multiple perspectives, facilitating morphology-guided surgical planning.

Initial anteroposterior pelvic radiographs demonstrated a displaced right acetabular fracture involving the posterior aspect of the acetabulum, with medial displacement of the femoral head. CT with multiplanar and three-dimensional reconstructions confirmed an associated fracture involving the anterior and posterior acetabular columns, with displacement of the posterior column while maintaining overall hip congruity. The fracture morphology indicated that adequate visualization of the posterior column and acetabular articular surface would be necessary for anatomical reduction (Figure 16).

Considering the fracture morphology, open reduction and internal fixation through the Ganz safe surgical dislocation approach with trochanteric flip osteotomy was performed. After the trochanteric flip osteotomy, safe surgical dislocation of the hip was achieved, allowing direct visualization of the posterior column and acetabular articular surface while preserving the blood supply of the femoral head. Anatomical reduction of the posterior column was performed under direct vision and stabilized using a six-hole reconstruction plate. The trochanteric osteotomy was subsequently reduced and fixed with two cancellous screws, restoring abductor continuity and hip stability.

**Figure 17:**
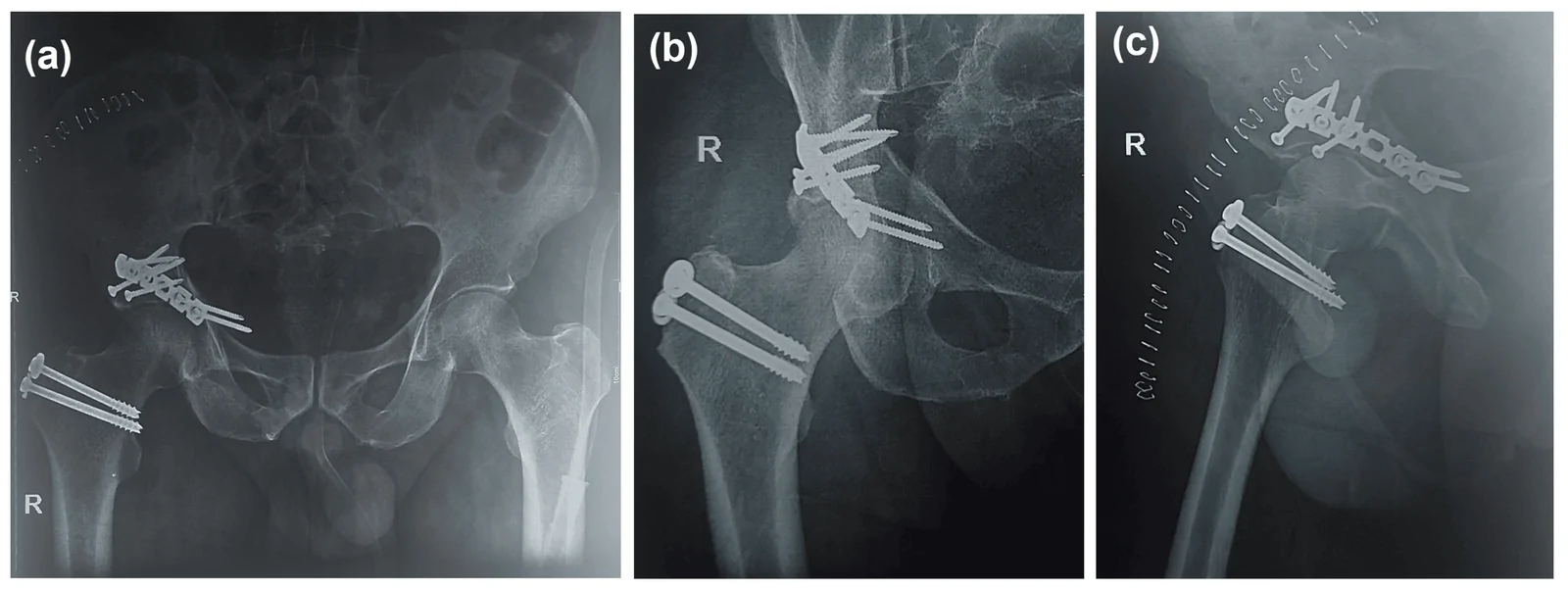
Case 6: Immediate postoperative assessment. (a) Immediate postoperative anteroposterior pelvic radiograph demonstrating anatomical reduction of the associated anterior and posterior column acetabular fracture, with stable reconstruction plate fixation of the posterior column and fixation of the trochanteric flip osteotomy using cancellous screws. (b) Immediate postoperative iliac oblique (Judet) radiograph demonstrating restoration of the posterior column and acetabular roof, with satisfactory reconstruction plate position. (c) Immediate postoperative obturator oblique (Judet) radiograph confirming anatomical reduction of the acetabular columns, restoration of hip congruity, and stable implant fixation.

Immediate postoperative anteroposterior, iliac oblique, and obturator oblique radiographs demonstrated satisfactory anatomical reduction of the acetabular fracture with restoration of hip congruity and stable implant position (Figure 17). The postoperative period was uneventful. The patient remained neurologically intact, and the surgical wound healed without complications. Protected non-weight-bearing mobilization and physiotherapy were initiated, followed by gradual rehabilitation.

Learning Point

Although the Ganz safe surgical dislocation approach is commonly used for femoral head fractures, it can also provide excellent exposure for selected acetabular fractures with predominant posterior column involvement. When carefully selected according to fracture morphology, this approach allows direct visualization of the articular surface, facilitates anatomical reduction, preserves femoral head vascularity through trochanteric flip osteotomy, and enables stable internal fixation.

## Discussion

The six cases presented in this series demonstrate that traumatic hip fracture-dislocations and complex acetabular fractures encompass a heterogeneous spectrum of injury patterns requiring individualized surgical strategies based on fracture morphology. Although all patients sustained high-energy trauma, the associated fracture configurations varied considerably and dictated the choice of surgical approach. The modified Stoppa approach was used for the central hip fracture-dislocation with anterior column and quadrilateral surface involvement; the Ganz safe surgical dislocation approach was selected for a Pipkin type II femoral head fracture and a selected associated anterior and posterior column acetabular fracture; and the Kocher-Langenbeck approach was employed for the posterior wall acetabular fracture associated with hip dislocation. Two additional patients with both-column acetabular fractures (AO/OTA types 62-C3 and 62-C1) were managed using a combined anterior and posterior approach and the modified Stoppa approach with the first window of the ilioinguinal approach, respectively. Despite the differences in fracture morphology and operative management, anatomical reduction and stable fixation were successfully achieved in all cases, highlighting the importance of individualized, morphology-guided surgical planning in optimizing radiological and functional outcomes (Table 1).

**Table 1:** Summary of clinical characteristics and surgical management of the six cases.

Variable	Case 1	Case 2	Case 3	Case 4	Case 5	Case 6
Age/Sex	26-year-old male	19-year-old male	25-year-old male	38-year-old female	22-year-old male	45-year-old male
Mechanism of injury	Fall from height	Road traffic accident	Road traffic accident	Fall from height	Fall from height	Road traffic accident
Injury pattern	Central hip fracture-dislocation with anterior column, quadrilateral surface, iliac blade, and undisplaced posterior column fractures	Posterior hip dislocation with Pipkin type II femoral head fracture	Posterior hip dislocation with posterior wall acetabular fracture	Both-column acetabular fracture (AO/OTA type 62 C3)	Both-column acetabular fracture (AO/OTA type 62 C1)	Associated anterior and posterior column acetabular fracture
Imaging	X-ray, CT, 3D CT	X-ray, CT, 3D CT	3D CT	CT, 3D CT	X-ray, CT, 3D CT	X-ray, CT, 3D CT
Surgical approach	Modified Stoppa	Ganz safe surgical dislocation	Kocher-Langenbeck	Combined anterior and posterior approaches	Modified Stoppa with first window of the ilioinguinal approach	Ganz safe surgical dislocation with trochanteric flip osteotomy
Special procedure	Corona mortis ligation	Trochanteric flip osteotomy	Direct posterior wall reduction	Sequential anterior and posterior column reduction	Quadrilateral surface buttressing with iliac wing fixation	Trochanteric flip osteotomy with direct posterior column visualization
Definitive fixation	Reconstruction plate, quadrilateral buttress plate, cortical screws	Herbert headless compression screws with cancellous screws for greater trochanteric osteotomy	Spring plates with posterior reconstruction plate	Pelvic brim reconstruction plate with posterior column fixation and quadrilateral buttress plate	Pelvic brim reconstruction plate with iliac wing fixation and anterior to posterior column screws	Six-hole reconstruction plate with cancellous screw fixation of the trochanteric osteotomy
Radiological outcome	Anatomical reduction with fracture union	Anatomical reduction with maintained hip congruity	Anatomical reduction with restoration of the posterior wall	Anatomical reduction with stable intraoperative fixation	Anatomical reduction with satisfactory postoperative implant position	Anatomical reduction with stable implant position
Functional outcome	Good recovery	Good recovery	Good recovery	Uneventful postoperative recovery	Uneventful postoperative recovery	Uneventful postoperative recovery

Traumatic hip fracture-dislocations are uncommon but severe injuries that usually result from high-energy trauma, most commonly road traffic accidents. Although posterior hip dislocations are the most frequent injury pattern, central and anterior dislocations occur less commonly and are often associated with acetabular or femoral head fractures, making management considerably more challenging [2,4,5,10-15]. The presence of associated fractures frequently precludes successful treatment with closed reduction alone and necessitates operative intervention to restore hip congruity and stability [4,5,10-12]. Therefore, careful evaluation of fracture morphology is essential before selecting the surgical approach.

Early recognition and prompt reduction remain the cornerstones of management for traumatic hip dislocations. Delayed reduction has consistently been associated with an increased risk of avascular necrosis of the femoral head, post-traumatic osteoarthritis, and inferior functional outcomes [4,5]. In addition to confirming reduction, computed tomography with three-dimensional reconstruction plays a crucial role in identifying associated acetabular fractures, femoral head fractures, incarcerated intra-articular fragments, and residual incongruity, thereby facilitating definitive surgical planning. In our series, CT imaging was indispensable in accurately defining fracture morphology and guiding operative decision-making in all patients.

The modified Stoppa approach has become an established anterior intrapelvic approach for fractures involving the anterior column and quadrilateral surface. Compared with the traditional ilioinguinal approach, it provides direct visualization of the anterior column, quadrilateral surface, and medial aspect of the posterior column while minimizing extensive soft-tissue dissection [16,17]. Liu et al. reported anatomical or satisfactory reduction in 96% of patients treated through the modified Stoppa approach and concluded that it provides excellent visualization of the anterior column and quadrilateral surface while maintaining a low complication rate [17]. Another important technical consideration highlighted by Liu et al. is meticulous identification of the corona mortis before quadrilateral surface dissection because uncontrolled injury to this vascular communication can result in significant intrapelvic hemorrhage [17]. In our first case, the fracture predominantly involved the anterior column and quadrilateral surface, making the modified Stoppa approach the most appropriate surgical exposure. Furthermore, prophylactic identification and ligation of the corona mortis before orthopedic exposure provided an additional safety measure during intrapelvic dissection and facilitated stable reconstruction of the acetabulum.

Pipkin fractures are uncommon injuries that usually occur following high-energy posterior hip dislocations and remain challenging because treatment must achieve anatomical reduction while preserving femoral head vascularity [18]. The Ganz safe surgical dislocation approach provides circumferential visualization of the femoral head and acetabulum through a trochanteric flip osteotomy while preserving the deep branch of the medial femoral circumflex artery, making it particularly suitable for Pipkin type I and II fractures [18]. Chen et al. also reported favorable clinical outcomes with surgical hip dislocation, emphasizing that stable fixation and preservation of femoral head blood supply are key determinants of successful treatment [18]. In our second case, the Ganz approach enabled direct visualization of the fracture, precise anatomical reduction, and fixation using Herbert headless compression screws, resulting in maintained hip congruity and satisfactory functional recovery.

Although the Ganz safe surgical dislocation approach is most frequently described for the management of femoral head fractures, its utility extends to selected acetabular fractures requiring circumferential visualization of the posterior acetabulum. In our sixth case, an associated anterior and posterior column acetabular fracture was managed through this approach using a trochanteric flip osteotomy. The surgical exposure provided excellent visualization of the posterior column and acetabular articular surface, facilitating anatomical reduction and stable plate fixation while preserving the vascularity of the femoral head. This case demonstrates that, in carefully selected patients, the Ganz approach represents a valuable alternative to conventional posterior approaches when fracture morphology necessitates direct visualization of the articular surface and posterior column.

Posterior wall acetabular fractures associated with posterior hip dislocation frequently produce hip instability and commonly require operative fixation. Computed tomography is essential for defining fracture morphology, identifying incarcerated intra-articular fragments, and planning definitive fixation. Anatomical reduction remains one of the strongest predictors of long-term functional outcome, while spring plates combined with posterior reconstruction plates provide stable fixation for comminuted posterior wall fractures by restoring posterior acetabular containment and hip stability [19]. These observations are consistent with our third case, in which an incarcerated posterior wall fragment prevented concentric reduction. Open reduction through the Kocher-Langenbeck approach allowed direct visualization of the posterior acetabulum, anatomical reduction of the fragment, and stable fixation using spring plates and a posterior reconstruction plate, resulting in restoration of hip congruity and good functional recovery.

Overall, the present case series reinforces that traumatic hip fracture-dislocations and complex acetabular fractures should not be managed using a single surgical approach. Instead, detailed CT-based evaluation and careful assessment of fracture morphology should guide the choice of surgical exposure and fixation strategy. In these six cases, selection of the operative approach according to the anatomical characteristics of the injury was followed by satisfactory radiological restoration and stable fixation. The diversity of fracture configurations included in this series further demonstrates that even the same surgical approach, such as the Ganz safe surgical dislocation approach, may have different indications depending on the underlying fracture morphology. These cases support the use of individualized, morphology-guided surgical planning in the management of complex traumatic hip fracture-dislocations and acetabular fractures.

This study has several limitations. First, it is a small case series of six patients from a single institution, and the fracture patterns were heterogeneous. Second, there was no comparison group, so the outcomes cannot be used to determine whether one surgical approach is superior to another. Third, standardized functional outcome scores were not used, and functional recovery was assessed clinically. In addition, exact follow-up intervals were not consistently available from the retrospective records, limiting assessment of long-term outcomes. Therefore, the findings should be interpreted as descriptive and educational rather than comparative. Larger multicenter studies with standardized functional assessments and predefined follow-up intervals are needed to further evaluate morphology-guided surgical strategies.

## Conclusions

Traumatic hip fracture-dislocations and complex acetabular fractures represent a heterogeneous group of injuries requiring individualized surgical management. In this six-case series, preoperative CT assessment of fracture morphology helped guide the selection of the surgical approach and fixation strategy for different injury patterns. The cases demonstrate the feasibility of morphology-guided surgical planning in achieving satisfactory radiological and clinical outcomes. However, the small sample size, heterogeneous fracture patterns, absence of a comparison group, and lack of standardized functional outcome measures preclude comparative conclusions regarding the superiority of any particular surgical approach. Larger studies with standardized outcome assessments and longer follow-up are needed to further evaluate this approach.
